# Influences of Experience and Visual Cues of Virtual Arm on Distance Perception

**DOI:** 10.1177/2041669519901134

**Published:** 2020-01-22

**Authors:** Zhen Yang, Jinlei Shi, Yi Xiao, Xiaojian Yuan, Duming Wang, Hongting Li, Weidan Xu

**Affiliations:** Department of Psychology, Zhejiang Sci-Tech University, Hangzhou, China; National Key Laboratory of Human Factors Engineering, China Astronauts Research and Training Center, Beijing, China; Department of Psychology, Zhejiang Sci-Tech University, Hangzhou, China; Hangzhou College of Commerce, Zhejiang Gongshang University, Hangzhou, China

**Keywords:** egocentric distance perception, visual cues, specific behavior, virtual reality, near space, far space, length of virtual arm

## Abstract

Egocentric distance perception refers to the perception of distance from a target to a perceiver, which is an important component of visual space perception. It is important to activities in virtual environments and influenced by several factors, such as action capacities and visual cues. However, few studies have investigated such aspects. Hence, Experiments 1 and 2 investigated the effect of using experience and visual cues, respectively, of virtual arms on egocentric distance perception in near and far spaces at equal, prolonged, and shortened lengths of a virtual arm. Results revealed that using experience and visual cues of the virtual arm had a significant effect on egocentric distance perception when the length of virtual arm was equal to the real arm and prolonged but not when shortened. The egocentric distance perception on the conditions of having using experience and virtual arm was most precise. The findings provide implications for the design and implementation of virtual body self-representation in virtual environments.

## Introduction

For several years, many researchers in the field of cognitive psychology have investigated depth perception (i.e., egocentric distance perception). As an important component of visual spatial perception, egocentric distance perception refers to the perception of distance from targets to perceivers. Several studies on the cues of egocentric distance perception such as binocular disparity, binocular convergence, lenticular accommodation, motion parallax, object overlay, linear perspective, atmospheric perspective, texture gradient, shadows, and size of familiar objects (Cutting & Vishton, 2012; Howard & Rogers, 1995) have been carried out. Recently, other studies have indicated that body cues, such as action capabilities, are significant for operators in determining distance (Proffitt & Linkenauger, 2013).

A study conducted by Proffitt, Bhalla, Gossweiler, and Midgett (1995) found that most individuals overestimate hill slopes after running, except for soccer players. This difference is due to the fact that soccer players train every day, and thus, their action capabilities are greater than regular people. Stefanucci and Geuss (2009) revealed that individuals with broad shoulders estimate the size of apertures to be smaller than people with narrow shoulder. Taylor, Witt, and Sugovic (2010) compared parkour experts, who are trained to kick off of walls and jump high, and height-matched novices. The results revealed that compared with novices, parkour experts perceived walls to be short when the wall afforded climbing for parkour experts but not the novices. In addition, other studies have found that softball players who hit better than other players view the ball as bigger (Witt & Proffitt, 2005). Tennis players who return more balls successfully than others view the net as lower (Witt & Sugovic, 2010). In summary, the aforementioned studies noted that the action abilities of participants have a vital effect on perception.

Theoretical basis of the influence of action capabilities on distance perception was based on Gibson’s theory of affordances (Witt & Riley, 2014). Gibson (1977) proposed that individuals perceive an environment in terms of provided opportunities for action, which are called affordances. They include whether a surface is sufficiently substantial, smooth, and of an orientation that can be walked upon; whether an object is of a size that can be grasped; or whether a wall is of a height that can be jumped over (Proffitt, 2013). The theory of affordances highlights that the choice of behavior judgment is dependent on the analysis of various behavioral possibilities based on various cues (Gibson, 1977). Recently, many studies on the influence of affordances on space perception have been carried out. For example, various scholars pointed out that participants estimated distance from targets using extended tools (e.g., pens and baton) or none when targets were placed out of arm’s reach. The results showed that estimated distance from targets is close when participants use tools (Costello et al., 2015; Osiurak, Morgado, & Palluel-Germain, 2012; Wagman & Malek, 2009; Witt & Proffitt, 2008; Witt, Proffitt, & Epstein, 2005). Simultaneously, other researchers investigated that the estimated distance from targets is only close when tools were sufficiently long to touch the objects (Witt & Proffitt, 2008).

However, to convincingly investigate the effect of arm’s reach on egocentric distance perception, manipulating arm’s length rather than changing the arm’s reach by using tools indirectly is necessary. The previous cases are not authentically manipulating arm’s reach. On the contrary, the arm has certain limitations in acting (Linkenauger, Bülthoff, & Mohler, 2015). In addition, these studies did not investigate the influence of the diminished reaching ability of the arm on egocentric distance perception. Thus, we pose the following question: “When the length of the arm is shortened, will the results be similar to that under the prolonged arm condition?” However, changing the morphology of the arm to manipulate its reach is difficult in the real world.

With the development of technology in virtual environments, conducting experiments previously deemed impossible in the real world has become convenient for researchers (Parsons, Gaggioli, & Riva, 2017; Zhou, Han, Liang, Hu, & Kuai, 2019). Using virtual reality (VR) technology, scholars can easily manipulate an experienced environment (Sanchez-Vives & Slater, 2005). Furthermore, using motion tracking systems, a virtual avatar can move in the same manner as that of a real body in real time. That is, participants can experience a virtual avatar and interact with other elements in virtual environments (Linkenauger et al., 2015). In addition, individuals can easily regard virtual limbs as their own in a virtual environment, and changing the avatar will not result in perception conflict. Kilteni, Normand, Sanchez-Vives, and Slater (2012) suggested that participants regarded virtual arms as counterparts when the length of the virtual arm was longer or shorter than that of the real arm. Such sense of ownership of this virtual body remains strong despite a drastic difference between the virtual and real limbs. Steptoe, Steed, and Slater (2013) investigated the ownership of a virtual body by installing a virtual tail for a virtual portrait. The results revealed that the participants experienced a strong sense of ownership of their virtual tails although they do not own the tails in the real word. Manipulating the characteristics of avatars in virtual environments is suitable for investigating their effect on egocentric distance perception.

Moreover, using tools to extend the reaching ability indirectly, participants find that adapting to a new ability is unnecessary due to the experience of using such tools in daily life. However, in virtual environments, we directly change the length of the virtual arm to manipulate the participants’ reaching ability. A significant difference in appearance is noted between the real and virtual arms. This result raises the issue as to whether the participants need using experience of virtual arm to adapt to the new reaching ability and thus apply the new altered metric as a perceptual scale.

Space was divided in “near space” and “far space” according to the reach of the operator arm. *Near space* is defined as the space within arm’s reach, whereas the opposite is true for *far space* (Lourenco & Longo, 2009). A stream of research revealed that distance between the target and participants also influences egocentric distance estimation. For example, Linkenauger et al. (2015) held that near and far spaces differed in terms of the efficacy of primary depth cues and other means. Armbrüster, Wolter, Kuhlen, Spijkers, and Fimm (2008) found that the distance estimation performance in near space was better than that in far space. Therefore, exploring the influence of using experience of the virtual arm on egocentric distance perception in near and far spaces when the arm was prolonged and shortened is necessary.

Visual cues of self-avatar have a significant effect on distance perception. Williams, Johnson, Shores, and Narasimham (2008) found that viewing a rendering of one’s static feet decreased the foreshortening of a bisection task within an Head Mounted Display based (HMD-based) virtual environment. In addition, Mohler, Creem-Regehr, Thompson, and Bülthoff (2010) found that participants who are exploring near space while seeing a fully articulated and tracked visual representation of themselves subsequently made accurate judgments of absolute egocentric distance to locations ranging from 4 m to 6 m away from their actual location than participants with no avatar cues. However, the height, arm span, and leg height of the avatar were scaled to match the physical dimensions of each participant in the cited studies, such that the reaching ability of the participants in VR was similar to that in real life. Therefore, when reaching ability is challenged by prolonging or shortening the virtual arm, whether the effect of the avatars’ visual cues on distance perception is the same as that when the virtual arm has equal length to the real arm remains unknown.

This study aims to investigate the effects of using experience and visual cues of the virtual arm on egocentric distance perception in near and far spaces when the length of the virtual arm was equal to the real arm and under prolonged and shortened conditions. Based on the literature, we hypothesize that the effects of using experience and visual cues of the virtual arm on egocentric distance perception differ in near and far spaces. Furthermore, the effects are also different when the length of the virtual arm varies.

## Experiment 1

### Objectives

This experiment aims to explore the influence of using experience (i.e., touching the target or not) of the virtual arm on the egocentric distance perception of targets in near and far spaces when the length of the virtual arm was equal to the real arm and under prolonged and shortened conditions.

### Methods

#### Participants

A total of 53 undergraduate and postgraduate students (26 females and 27 males, aged 18 to 25 years) were recruited as participants for this experiment. All participants had normal or corrected-to-normal vision.

#### Materials

The experiment was conducted in a virtual room. The size and location of the virtual room were the same as those of a laboratory. We placed a virtual chair in a virtual room, and its position was the same as that of a real chair in a laboratory. The materials included practical and formal experimental materials.

Practical materials refer to materials used in the practical phase. Specifically, virtual objects had different distances (30, 45, 60, 75, and 90 cm) from the participants in the virtual space. Several objects can be reached by the virtual arm (in near space) but other objects could not (in far space). The position, orientation, and rotation of the virtual arm were controlled by the participants by moving the hand grip.

Formal experimental materials are identical to those in the practical phase, except that the distances of the target objects from the participants were 12 cm longer or shorter than the actual length of the virtual arm. This distinction indicates that the objects were placed in far and near spaces separately.

#### Experimental equipment

An ASUS high-performance laptop with an Intel Core i7-6700HQ processor, 2.6 Hz dominant frequency, and a GTX 1070 independent graphics card of 6 GB display memory was used. HTC Vive HMD consisted of a VR glass, two hand grips, and two positioners. Two eyes were supplied with one display screen with a resolution of 1,080 × 1,200 pixels and a refresh rate of 90 Hz.

A bracket on the table was used to fix the participants’ head and control the height of the participants’ eyes (control participants’ viewing angle). One table and one chair were placed in the laboratory. The chair can be rotated, and its height can be freely adjusted. The location of the chair was similar to that of the virtual chair.

We used C# language and Unity game engine to develop the experiment program with Visual Studio 2017. We supplied Application Programming Interface (API) function by calling the Software Development Kit (SDK) of the HMD to obtain the coordinates of the helmet and hand grip. After obtaining the position of the hand grip, we updated the location of the virtual hand and arm in the virtual screen in real time according to the location parameter of the hand grip. Hence, the participants were able to control their virtual hand and arm in the virtual environment by moving their hand grips. Moreover, the experimental program included a distance adjustment module. A virtual glide bar was dragged via the hand grip. In addition, an experimental program automatically recorded the participants’ information and experimental data.

#### Experimental variables and design

A three-factor mixed design was used for the experiment. The length of the virtual arm (equal to the real arm, 30% longer, and 30% shorter than the real arm) was considered the between-subject factor. The distance of the objects from the participants (12 cm shorter than the length of the virtual arm [in near space] and 12 cm longer than the length of virtual arm [in far space]) and using experience of the virtual upper limb (touching the target objects or not) were treated as within-subject factors. The task sequence on each condition was balanced by a Latin square design.

The dependent variable comprised estimates of the egocentric distance from the participants to objects. The participants were asked to adjust the length of a virtual glide bar until they felt confident that the length of the glide bar matched the distance from the objects. The length of the glide bar pertains to the estimated value of egocentric distance. The glide bar was located 90° on the left side of the participants, and the direction of the participants facing the target object differed from that of the participants facing the glide bar to prevent such participants from looking for reference objects to assist in estimating distance.

#### Experimental task

**Figure 1.**
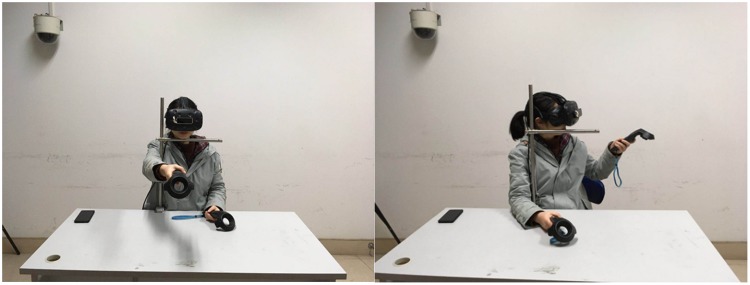
Experiment scene. The left figure was the scene in which the participant touched the target object in the VR environment. The right figure was the scene in which the participant adjusted the length of the glide bar, which indicated the estimated distance.

All experimental tasks were completed in the virtual environment. In the practice phases and under the condition of having using experience, the participants held and placed the hand grips in front of their chests. When they pulled the hand grip trigger, the target object appeared in front of them. They were told to reach and touch the virtual object by controlling the hand grip. The object disappeared as soon as the participant pulled the trigger again. If the object was extremely far for the participants to reach, then they were instructed to point to the object and pull the trigger instead. Then, the participants were asked to turn left by 90° and face the glide bar. They matched the length of the glide bar to the distance between the participants and virtual objects by controlling the hand grips with their left hands (Figure 1). Under the condition of having no using experience, the participants were told to hold the hand grips by their right hands and make them flatwise on the desk from beginning to end.

In the formal experiment, the task was the same as that in the practical phase.

#### Procedure

After the participants entered the laboratory, they sat on the chair with a fixed position. Then, the participants were asked to place their chins on the bracket (which was fixed to the table with set position and height) and adjust the height of the chair until they felt comfortable. The experimenter explained the operation method of the hand grip and assisted the participants in wearing the HMD. After wearing, the participants held and placed the hand grips in front of their chests.

Instructions were presented to the participants, and the experimenter explained it on the side (the picture that the experimenter saw from the computer display was identical to that in the VR). The participants straightened their right arms and pulled the trigger. The program automatically recorded the position of the hand grip and transformed it to the length of the real arm. This mechanism is due to the varied arm’s length of the participants. Hence, in the experiment, each participant’s length of arm was recorded as a parament in the experimental program, such that the program can set the length of the virtual arm and distance of objects for each participant separately according to the parament. As a result, the virtual arm’s length of the different participants varied, and the locations of the target object for each participant were also different. In the statistics phase, we used an algorithm to transform the arm’s length of each participant to 60 cm, which indicates that the virtual arm with a 60 cm-length was once a real arm. The detailed formula is presented as follows:

$$\text{Estimated}\;\text{distance}=\frac{60e}{n}$$

where *e* is the original estimated distance, and *n* denotes the real arm’s length of the participant. The same method was used for Experiment 2.

The participants were then divided into three groups according the lengths of the virtual arm. In each group, the experiment was divided into two blocks according to with or without using experience. The sequence of the two blocks was random. Each block contained two phases (i.e., practical and formal experiment phases). For the practical phase, each distance was presented three times, with random sequences for each distance. The experimenter switched the program into the formal experiment after confirming that the participants understood the tasks of the experiment. Each distance was presented three times with a random sequence.

### Results

All data were analyzed using SPSS 13.0 (Table 1).

**Table 1.** Mean of Estimated Egocentric Distance and Standard Error in Near and Far Space With or Without Using Experience When the Length of Virtual Arm Was Shortened, Equal to Real Arm and Prolonged.

Shortened arm				Equal length arm				Prolonged arm			
Without using experience		With using experience		Without using experience		With using experience		Without using experience		With using experience	
Near space	Far space	Near space	Far space	Near space	Far space	Near space	Far space	Near space	Far space	Near space	Far space
37.53 ± 3.35	61.25 ± 4.13	37.27 ± 2.09	61.01 ± 2.41	60.47 ± 3.45	80.12 ± 4.25	52.93 ± 2.15	74.84 ± 2.47	78.97 ± 3.35	101.58 ± 4.13	65.69 ± 2.09	89.55 ± 2.41

*Note.* The unit of estimated egocentric distance is cm.

A three-way repeated-measures analysis of variance (ANOVA) was conducted with using experience and distances from target object as within-subject independent variables. The length of the virtual arm was assigned as the between-subject independent variable, and the estimates of egocentric distance were considered the dependent variable. The main effects for with and without using experience, *F*(1, 50) = 15.39, *p* < .001, *η*_p_^2^ = 0.24; distance of objects, *F*(1, 50) = 651.35, *p* < .001, *η*_p_^2^ = 0.93; and length of the virtual arm, *F*(2, 50) = 44.29, *p* < .001, *η*_p_^2^ = 0.64 were observed. Post hoc testing revealed that the estimate of egocentric distance without using experience was significantly farther than that with using experience (*p*s < .001). The estimate of egocentric distance under the prolonged arm condition was significantly farther than that under the equal length condition (*p*s < .001). The estimate of egocentric distance under the equal length arm condition was significantly farther than that under the shortened arm condition (*p*s < .001). Moreover, interaction between with and without using experience and length of the virtual arm was significant, *F*(2, 50) = 4.87, *p* < .05, *η*_p_^2^ = 0.16.

**Figure 2.**
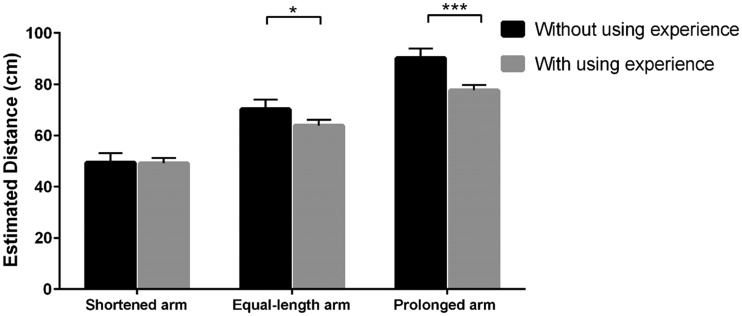
Estimated distance with or without using experience when the length of the virtual arm is shortened, equal to the real arm, and prolonged. *p < .05; ***p < .001.

We further conducted simple effect analysis, which revealed that when the length of the virtual arm was shortened, with or without using experience had no effect on the estimated egocentric distance. However, when the length of the virtual arm was equal to the real arm and prolonged, the estimated egocentric distance without using experience was farther than that with using experience (*p*s < .05; Figure 2).

**Figure 3.**
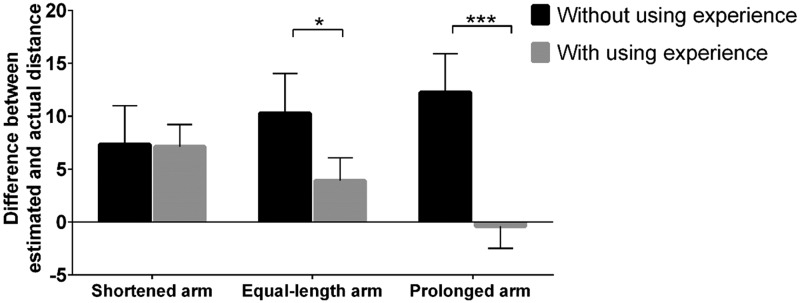
Difference between estimated and actual distances with and without using experience when the length of the virtual arm was shortened, equal to the real arm, and prolonged. *p < .05; ***p < .001.

To probe the influence of length and using experience of the virtual arm on the estimated precision, we also conducted another ANOVA with distance from objects, length and using experience of virtual arm as independent variables, and the difference between the estimated and actual distances as the dependent variable. Main effects for distance of objects and the length of virtual arm were not significant. The main effect for with and without using experience was significant, *F*(1, 50) = 15.15, *p* < .001, *η*_p_^2^ = 0.23. Post hoc testing revealed that the estimated precision with using experience was greater than that without using experience (*p*s < .001). Furthermore, the interaction between using experience and length of the virtual arm was also significant, *F*(2, 50) = 4.83, *p* < .05, *η*_p_^2^ = 0.16. Simple effect analysis revealed that when the length of the virtual arm was shortened, no significant difference was observed for neither with nor without using experience. In addition, when the length of the virtual arm was equal to the actual or prolonged arm, the estimated precision was greater for with using experience of the virtual arm than that without using experience (*p*s < .05; Figure 3).

## Experiment 2

### Objectives

This experiment aims to explore the influence of visual cues (i.e., virtual arms, hands, and solid circles) on the egocentric distance perception of targets in near and far spaces when the length of the virtual arm was equal to the real arm, prolonged, and shortened.

### Methods

#### Participants

A total of 60 undergraduate and postgraduate students (30 females and 30 males, aged 18–25 years) were recruited as participants for this experiment. The participants had normal or corrected to normal vision.

#### Materials

**Figure 4.**
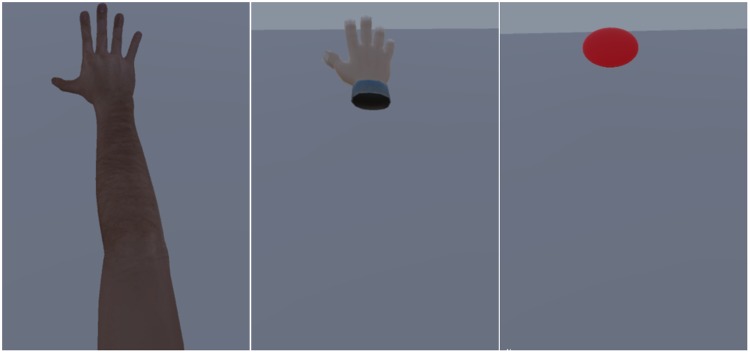
Different visual cues of the arm in the VR environment (left: entire virtual arm; middle: virtual hand only; right: virtual solid circle).

Virtual upper limbs included three forms, namely, virtual arm, virtual hand, and virtual solid circle. Virtual arm refers to an entire virtual arm (Figure 4, left panel). The virtual hand refers to only one virtual hand (Figure 4, middle panel). A virtual solid circle refers to an abstract solid circle that replaces the virtual hand (Figure 4, right panel). The other materials were the same as those in Experiment 1.

#### Experimental equipment

The equipment used in this experiment was identical to that used in Experiment 1.

#### Experimental variables and design

A three-factor mixed design was used for the experiment. The length of the virtual arm (equal to the real arm and 30% longer and 30% shorter than the real arm) was used as the between-subject factor. The distance of objects from the participants (12 cm shorter than the length of the virtual arm [in near space] and 12 cm longer than the length of the virtual arm [in far space]) and visual cues of the virtual arm (virtual arms, hands, and solid circles) were considered within-subject factors. The task sequence on each condition was balanced by a Latin square design.

The dependent variable was identical to that in Experiment 1.

#### Experimental task

The experimental task was identical to that under the condition of having using experience in Experiment 1.

#### Procedure

The procedure was identical to that in Experiment 1.

### Results

All data were analyzed using SPSS 13.0 (Table 2).

**Table 2.** Mean of Estimated Egocentric Distance and Standard Error in Near and Far Space With Various Visual Cues When the Length of Virtual Arm Was Shortened, Equal to Real Arm and Prolonged.

Shortened arm						Equal-length arm						Prolonged arm					
Virtual solid circle		Virtual hand only		Virtual arm		Virtual solid circle		Virtual hand only		Virtual arm		Virtual solid circle		Virtual hand only		Virtual arm	
Near space	Far space	Near space	Far space	Near space	Far space	Near space	Far space	Near space	Far space	Near space	Far space	Near space	Far space	Near space	Far space	Near space	Far space
39.01 ± 2.52	61.45 ± 3.27	37.00 ± 2.37	61.01 ± 3.32	38.61 ± 2.12	61.19 ± 2.69	63.64 ± 2.52	85.25 ± 3.27	58.29 ± 2.37	81.69 ± 3.32	54.79 ± 2.12	77.27 ± 2.69	78.38 ± 2.52	101.91 ± 3.27	72.77 ± 2.37	98.54 ± 3.32	69.41 ± 2.12	94.04 ± 2.69

*Note.* The unit of estimated egocentric distance is cm.

A three-way repeated-measure ANOVA was conducted with visual cues of the virtual arm, and distances from the target object as within-subject variables, the length of the virtual arm as the between-subject variable, and estimates of egocentric distance as the dependent variable. The main effect for visual cues, *F*(2, 114) = 8.60, *p* < .01, *η*_p_^2^ = 0.13; distance of objects, *F*(1, 57) = 834.25, *p* < .001, *η*_p_^2^ = 0.94; and length of virtual arm, *F*(2, 57) = 68.52, *p* < .001, *η*_p_^2^ = 0.71 were observed. Post hoc testing revealed that the estimate of egocentric distance under the virtual solid circle condition was significantly farther than that under the virtual arm and hand conditions (*p*s < .01). No significant difference was observed between the virtual hand and arm. The estimate of egocentric distance under the prolonged arm condition was significantly farther than that under the equal length condition (*p*s < .001). The estimate of egocentric distance under the equal length arm condition was significantly farther than that under the shortened arm condition (*p*s < .001). Moreover, no interaction was significant.

**Figure 5.**
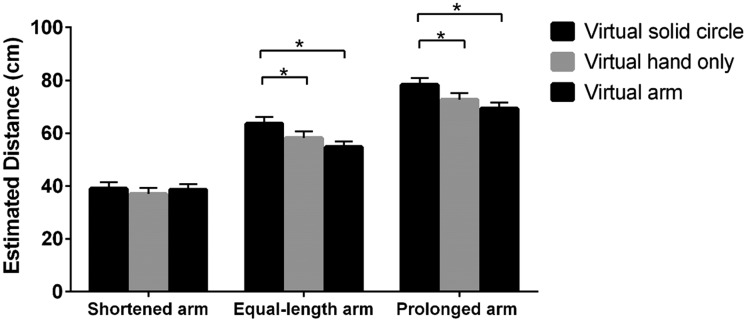
Estimated distance with various visual cues in near space when the length of virtual arm was shortened, equal to the real arm, and prolonged. *p < .05.

**Figure 6.**
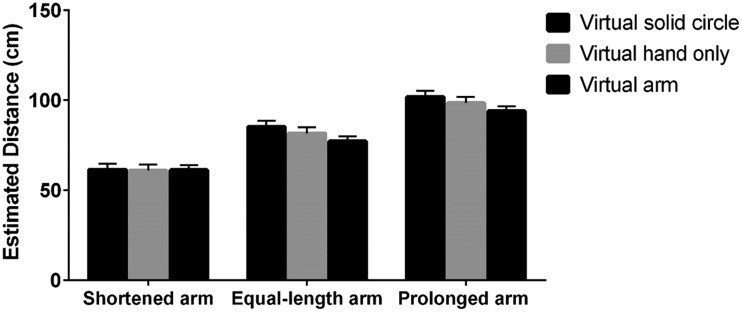
Estimated distance with various visual cues in far space when the length of virtual arm was shortened, equal to the real arm, and prolonged.

To further analyze the effect of visual cues and length of virtual arm on egocentric distance perception, we conducted a two-way ANOVA in near and far spaces, respectively. ANOVA in near space revealed that the main effect of the length of the virtual arm, *F*(2, 57) = 80.14, *p* < .001, *η*_p_^2^ = 0.74, and visual cues, *F*(2, 114) = 12.56, *p* < .001, *η*_p_^2^ = 0.18, were significant. In addition, the interaction between the length of the virtual arm and visual cues was significant, *F*(4, 114) = 2.62, *p* < .05, *η*_p_^2^ = 0.08. Simple effect analysis revealed that the estimate of egocentric distance under the virtual solid circle condition was significantly farther than that under the virtual arm and hand conditions when the length of virtual arm was equal to the real arm and prolonged (*p*s < .05). No significant difference was noted when the length of the virtual arm was shortened (Figure 5). ANOVA in far space revealed that the main effect of the length of the virtual arm, *F*(2, 57) = 51.59, *p* < .001, *η*_p_^2^ = 0.64, and visual cues, *F*(2, 114) = 4.78, *p* < .05, *η*_p_^2^ = 0.08, were significant. Although the interaction between the length of the virtual arm and visual cues was nonsignificant, a tendency to be significant exists. Simple effect analysis revealed that estimated egocentric distance under the virtual solid circle condition was farther than that under the virtual arm condition when the length of the virtual arm was equal to the real arm (*p* = .069) and prolonged (*p* = .075). Furthermore, no significant difference was observed when the length of the virtual arm was shortened (Figure 6).

**Figure 7.**
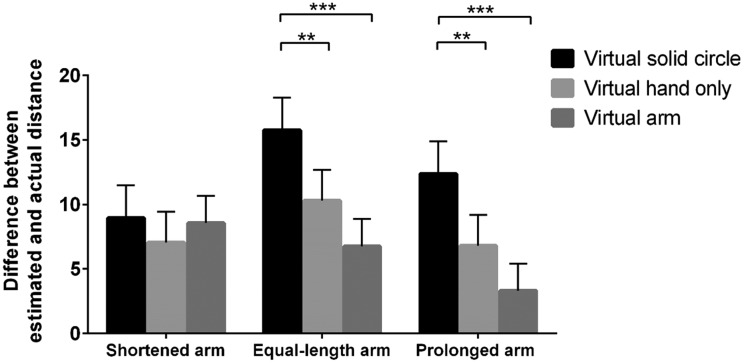
Difference between estimated and actual distances with various visual cues in near space when the length of virtual arm was shortened, equal to the real arm, and prolonged. **p < .01; ***p < .001.

**Figure 8.**
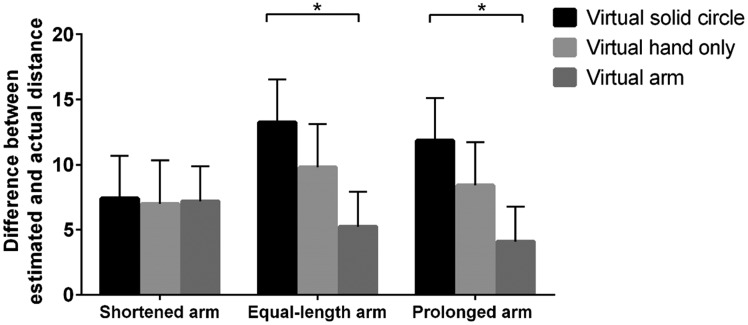
Difference between estimated and actual distances with various visual cues in far space when the length of the virtual arm was shortened, equal to the real arm, and prolonged. *p < .05.

We also conducted another ANOVA to probe the influence of distance from objects, length, and visual cues of the virtual arm on estimated precision, with the difference value that the estimated distance subtracted from the actual distance as the dependent variable. The main effect for visual cues was observed, *F*(2, 114) = 8.61, *p* < .001, *η*_p_^2^ = 0.13. Post hoc testing revealed that the estimated precision on the condition of the virtual arm was greater than that on the conditions of the virtual solid circle and hand (*p*s < .05). No other main and interaction effects were observed. To further analyze the effect of visual cues and length of the virtual arm on egocentric distance perception, we conducted two-way ANOVA in near and far spaces, respectively. ANOVA in near space revealed that the main effect for visual cues was significant, *F*(2, 114) = 12.73, *p* < .001, *η*_p_^2^ = 0.18, and the interaction effect between visual cues and length of the virtual arm was also significant, *F*(4, 114) = 2.67, *p* < .05, *η*_p_^2^ = 0.09. Simple effect analysis revealed that when the length of the virtual arm was equal to the real arm or prolonged, the estimated precision on the condition of the virtual arm was greater than that of the virtual solid circle and hand conditions (*p*s < .01). No significant difference was observed between these conditions when the length of the virtual arm was shortened (Figure 7). ANOVA in the far space gained a result similar to that in near space. The main effect for visual cues was significant, *F*(2, 114) = 4.68, *p* < .05, *η*_p_^2^ = 0.08. Although the interaction between the length of the virtual arm and visual cues was non-significant, a tendency to be significant exists. Simple effect analysis revealed that when the length of the virtual arm was equal to the real arm or prolonged, the estimated precision under the virtual solid circle condition was worse than that under the virtual arm (*p*s < .05) and hand conditions (*p*s < .1). No significant difference was observed when the length of the virtual arm was shortened (Figure 8).

## General Discussion

The present study found that using experience and visual cues of virtual arm had a significant effect on egocentric distance perception when the length of the virtual arm was equal to the real arm or prolonged but not when shortened. The estimated distance on the condition of with using experience was more precise than that without using experience. The estimated distance on the condition of the virtual arm was more precise than that on the virtual arm and virtual solid circle conditions.

### Influence of Using Experience of Virtual Arm on Egocentric Distance Perception

Experiment 1 explored the influence of using experience of the virtual arm (i.e., touching the target object) on egocentric distance perception. Results showed that using experience of the virtual arm had no effect on distance perception on the shortened arm condition. Distance estimation without using experience was farther than with using experience when the arm was prolonged or equal to the real arm.

The participants estimated distance more precisely with using experience than without using experience. This result is consistent with the findings of other studies. For example, Linkenauger et al. (2015) determined that no significant difference in distance estimation existed between prolonged and shortened arms without action, but significant differences with actions were observed. Ramenzoni, Riley, Shockley, and Davis (2008) found that individuals were capable of accurately predicting changes in their ability to jump after donning ankle weights by walking for 2 minutes. Perceived action capabilities were likely used as perceptual metrics. If the new action capability was unspecified due to the lack of movement experience, then perceivers had no new metric with which to rescale their distance perception. Experiment 1 demonstrated this demand for experience to rescale distance perception.

In addition, using experience had no effects on estimated distance when the arm was shortened. Gourlay, Lun, Lee, and Tay (2000) determined that skills can be transferred from a virtual environment to the real world. This finding indicates that the experience in VR influences such behavior in the real world. Similarly, experiences in the real world can influence the behavior in VR (Rosenberg, Grantcharov, Bardram, & Funch-Jensen, 2003). Thus, people can experience the extension of an arm’s reach through different tools in daily life, such as picking up clothes with a clothesline pole. The participants adapted to the prolonged arm in the experiment. However, no situation was observed in which the arm’s reach was limited in daily life; thus, the participants may not adapt to the shortened arm in the VR environment. Another possible interpretation is that the 30% shortened virtual arm might be too short to result in an effect. Increasing of the length of the virtual arm will increase the estimated distance correspondingly, and the difference of the estimated distance between with and without using experience might be more significant. Therefore, when the length of the virtual arm is shortened by 30%, the difference of the estimated difference between two conditions might be minor so as not to result in an effect. To verify this notion, setting added levels of shortened virtual arm in the future studies is necessary to explore the quantitative effect of the length of the virtual arm length on distance perception.

### Influence of Visual Cues of Virtual Arm on Egocentric Distance Perception

Experiment 2 explored the influence of visual cues on egocentric distance perception based on the conditions of equal length, prolonged, and shortened arms. Results indicated that visual cues had significant effects on distance estimation when the virtual arm was prolonged and had equal length. The distance estimation was farther when the virtual cue was abstract (solid circle) than that when the virtual cues were the arms and hands.

Thus, the visual cue of the virtual arm is a significant factor that can influence distance estimation after prolonging it. The estimated distance on the condition of the virtual arm is also the most precise, whereas the opposite is true for the virtual solid circle condition. An alternative explanation for these results can be drawn from body ownership. Many studies revealed that the strength of body ownership is dependent on the degree of morphological similarity between a real biological arm or hand and the external object to be incorporated (Armel & Ramachandran, 2003; Ehrsson, Spence, & Passingham, 2004; Tsakiris, Carpenter, James, & Fotopoulou, 2010; Tsakiris, Costantini, & Haggard, 2008; Tsakiris & Haggard, 2005). These studies showed that the illusion of ownership diminishes when the external object does not resemble their own body. Moreover, studies indicated that the ownership of body influences perception in VR. Alshaer, Regenbrecht, and O’Hare (2017) found that participants who used HMD have a stronger sense of ownership and better perceptual measure compared with participants who only use a monitor. In the present study, the virtual arm provided detailed information about their body, and thus, the participants had a strong sense of ownership over their arms. This sensation of ownership resulted in the best distance estimation. However, when the visual cue was an abstract solid circle, the participants experienced difficulty regarding the virtual solid circle as their counterpart due to the lack of detailed information. In this situation, the participants cannot precisely estimate distance according to the visual cue.

In addition, the visual cues had no effect on the estimated distance when the arm was shortened. This finding can be explained by the same reason previously proposed, that is, no situation occurred in which the reach of the arm was limited in daily life or that the 30% shortened arm was too short to result in an effect.

Although this study was carefully prepared, several limitations remain. First, we only explored distance perception at one point in near or far space. Future studies should consider other locations of targets in near or far space to investigate the quantitative effect of various distances on distance perception. Moreover, this study only set a 30% shorter or longer length of the real arm as arm conditions. Hence, the prolonged or shortened degree of the virtual arm should also be considered to explore their impact on distance perception in future studies. Finally, we only tracked the location and orientation of the participants’ real hand to update the virtual hand in the virtual environment due to technology limitations. The motion of real arms’ other locations was untracked such that certain differences were observed between the motions of the real and virtual arms.

In summary, the research demonstrates the influence of visual cues and using experience of the arm on the estimated distance when the arm is prolonged and has equal length to the real arm. In addition, no effect is observed when the arm is shortened. These findings add to the knowledge on how participants estimate distance through visual cues and using experience. They also have implications for the design and implementation of virtual body self-representation in virtual environments. Moreover, visual cues and using experience of the arm have no effect on egocentric distance perception when the arm is shortened. Thus, further investigations on the influence of shortened arms on VR perception may be of interest.
